# A Biophysical Approach to Functional Gallbladder Disorder Leads to the Hypothesis That Salmonella Plays a Relevant Role in the Etiology of This Disease: A Retrospective Observational Pilot Study

**DOI:** 10.7759/cureus.50398

**Published:** 2023-12-12

**Authors:** Hendrik Jan Hummelen

**Affiliations:** 1 Integrative Medicine, Practice Slunterzicht, Ede, NLD

**Keywords:** biliary dyskinesia, bioresonance, biophysical medicine, primary care, medically unexplained physical symptoms, salmonella, acalculous cholecystitis, functional gallbladder disorder

## Abstract

Introduction

Between 2014 and 2021, 41 patients with somatically inexplicable gastrointestinal complaints presented to the complementary and alternative medicine (CAM) practice of the author. Of these patients, 33 underwent diagnostic and therapeutic procedures in conventionally oriented practices and academic hospitals. The remaining eight participants directly reported the authors’ practices. Conventional interventions did not lead to sufficient improvement in these patients.

Patients and methods

This study aimed to characterize the presentation and treatment results of 41 patients selected using biophysical diagnostic technology (BICOM bioresonance; Regumed GmbH, Planegg, Germany) with the working diagnosis of chronic Salmonella Cholecystitis (CSC). A retrospective observational analysis of the records of these patients was performed to provide a better understanding of their clinical picture.

Results

After an initial treatment period of an average of 7.5 weeks, the "end of treatment" (EOT) score could be determined. With an average of +/- 4 bioresonance sessions, 66% of the patients had a reasonable to a good reduction in complaints. This number increased after additional bioresonance therapy of the patients with comorbidities to a follow-up score (FU) of 86%.

Conclusions

The findings of this pilot study support the hypothesis that CSC is a well-defined clinical entity and may even coincide with the clinical picture of functional gallbladder disorder (FGD). Both can be considered as energetically informative syndromes. The study suggests that biophysical medicine may be a viable option in the diagnosis and treatment of CSC. A prospective follow-up study in an integrated setting is needed to provide more insight into these diseases.

## Introduction

Physicians with a consultative practice for complementary and alternative medicine often encounter patients who have been ill for a long time without proper diagnosis or therapy. Most of these patients have already visited their family physicians several times, and some have completed diagnostic procedures and even therapies in general hospitals or academic centers without satisfactory results. Most patients in complementary and alternative medical (CAM) practices are not referred to by their GP but make an appointment on their own initiative [1-2]. Thus, patients with medically unexplained physical symptoms (MUPS) form a substantial proportion of the population in these practices [3]. Somewhat generalizable, it could be stated that an important part of the work of complementary doctors consists of the diagnosis and treatment of what is known as 'unexplained complaints'. Over the years, the author has regularly encountered patients with vague and poorly understood upper abdominal complaints that aroused his special interest. One such diagnosis that could not be established with a biophysical test method is that of chronic Salmonella cholecystitis (CSC). For this disease, biophysical interventions were usually successful, wherein the term 'biophysical' implies the application of physical principles in biological sciences. Within complementary medicine, this term specifically relates to the concept that electromagnetic processes play a vital role within organic systems [4].

Background and aims 

Research Question

What are the changes in complaints on a score scale after a number of biophysical treatments in a group of patients who present with persistent upper abdominal complaints in combination with a Salmonella infection of the gall bladder according to criteria from biophysical medicine?

Research Aim

The aim is to describe the symptomatology, treatment course, and outcomes of a defined group of patients with persistent upper gastrointestinal symptoms, as well as a biophysical diagnosis of CSC. Salmonella is a major cause of diarrhea worldwide, with a global burden of about 94 million cases (mostly foodborne) and 155,000 deaths yearly for nontyphoidal Salmonella gastroenteritis [5,6]. In the Netherlands, infections by non-typhus Salmonella occur regularly, usually in the form of food poisoning. In approximately 70% of cases, this infection is caused by either Salmonella enteritidis or Salmonella typhimurium. Many animals, including farm animals, are reservoirs of human-relevant Salmonella. In 2019, the total number of cases of acute gastroenteritis caused by Salmonella in the Dutch population was estimated to be almost 26,000 [7]. In addition to environmental contact and person-to-person transmission, an estimated 55% of Salmonella infections are transmitted through the consumption of contaminated animal products (e.g., meat, fish, or eggs) [8]. Microorganisms can infiltrate the lymphatic system, enter the bloodstream, and reach other organ systems along this route, including the gallbladder, for which Salmonella species have a special affinity [9]. This provokes an inflammatory process with characteristic symptomatology, possibly corresponding to the clinical diagnosis described as functional gallbladder disorder (FGD) [10], previously referred to under different names such as gallbladder spasms, chronic acalculous cholecystitis, chronic acalculous gallbladder dysfunction, and gallbladder dyskinesia. The core symptoms of FGD include recurrent pain and discomfort in the upper abdomen, nausea, and vomiting. It does not usually involve structural pathologies, gallstones, or other organic causes of biliary pain. Cholecystokinin-stimulated scintigraphy (CSS) may yield a low ejection fraction of the gallbladder; however, a normal ejection fraction does not exclude FGD. The values predicted by these tests are controversial [11], and the aetiology of FGD is still unclear [12-14]. The hypothesis is that disturbed biliary mobility plays a central role in the pathogenesis of FGD. In summary, the diagnosis of FGD was based on the exclusion of organic pathology and symptom-based ROME IV criteria [15,16]. The purpose of using these criteria is to answer difficult questions using a consensus methodology about a group of gastrointestinal disorders that have little scientific evidence to understand their aetiology, pathophysiology, and treatment. The diagnosis of FGB is complex and time-consuming as other gastrointestinal pathologies must be excluded. However, FGD is rarely diagnosed. In a cross-sectional survey of 5,931 adults with Rome IV-diagnosed Functional Gastrointestinal Disorders (FGIDs), only 0.2% met the defined criteria for FGD [16]. Since this condition may resolve spontaneously, data on the natural history of FGD are limited. FGD is a common indication for surgery and accounts for 2%-5% of adults and up to 10% of children [17-19]. An expectant treatment approach, combined with analgesics and spasmolytics, is recommended as an alternative.

Study Design

The research question was answered through a retrospective observational study.

Introduction of the Principles of Biophysical Medicine and Bioresonance Therapy

The term 'biophysical' [20-22] implies the application of physical principles in biological sciences. In complementary medicine, the term specifically relates to the concept that electromagnetic processes play a vital role in organic systems. The basic concept of biophysical medicine is that an organism has a biophysical (electromagnetic) regulatory system that is placed in a hierarchy above the humoral and neuronal levels of regulation. The disease is thought to originate from dysregulation of this regulatory system. The treatment of the disease focuses on this issue. The model used in biophysical medicine considers a patient as a biological system that is disrupted to such an extent that the body's own regulation is no longer capable of adequate counter-regulation to eliminate the pathological burden [23-25]. When this counter-regulation of the self-healing process fails, often under chronic conditions, active intervention is required to restore the body within its control range. In complementary and alternative medicine, the term 'self-regulation' is commonly used as a general guiding principle for the healing process. Within the CAM disease is regarded as a failure of the self-healing capacity of the biological system. Bioresonance therapy aims to assist biological systems in this regulation. This therapy and other natural healing methods are part of the field of empirical medicine. The fundamentals of the bioresonance therapy paradigm are briefly summarised below and are consistent with biophysical findings [26-29].

Immaterial Information Transfer

The first concept is the existence of immaterial information transfer between the cells of a living organism. In 1927, the Russian biophysicist Gurwitsch [30] demonstrated that electromagnetic radiation plays a vital role in plant growth. He spoke of "mitogenic radiation" as the guiding information for plants. The influence of weak static and low-frequency alternating magnetic fields on biological systems has been extensively reviewed by the Russian scientist Zahdin [31], who discovered that ultrasmall magnetic intensities are biologically significant and suggested that EM signalling is endogenous to cell regulation. Consequently, he stated that the remarkable effectiveness of the EM resonance treatment reflected a fundamental aspect of biological systems. The German physicist Popp [32,33] played an important role in the development of biological electromagnetism. After establishing that electromagnetic regulation of biological processes relies on signals with extremely small amplitudes, he introduced the term 'ultraweak'. These energetic carriers for cell communication, coined by Popp as ‘biophotons’, are found in the spectral range of visible and infrared light. This observation that human body cells receive and emit electromagnetic signals in the form of specific oscillation patterns for communication and regulation purposes foreshadowed the therapeutic application of electromagnetic signals in medicine.

Electromagnetic Signal Disharmony

The second concept is the assumption that information with a different frequency pattern or interference field, such as an invader of microbial, toxic, or energetic origin, may disrupt the regulation of an organism in such a way that it can lead to illness. This means that signal disharmony at the level of electromagnetic regulation can be considered the biophysical basis of the disease [24]. Consequently, the treatment of the disease primarily involves recovery from signal disharmony [32,33].

Information Storage

The third concept, information storage, is an important concept in biophysical medicine [34-37]. It is assumed that the therapeutic information can be electronically copied to a carrier substance and stored. Liquids, such as water and alcohol, have bipolar characteristics. Once exposed to a bioinformative energy field, the configuration of complex spatial cluster structures in the fluid is considered to store information of highly complex quality for a longer period. According to these principles, this also concerns pathological information of any kind, which can be stored in the body and strains the regulatory system. It is often seen that pathological information is no longer detectable as a material state, but as the so-called informative footprint, a characteristic frequency pattern that matches with the pathogenic substance or invader. The assumption is that, by administering targeted bioresonance therapy, the cluster structures of molecules containing this pathological oscillation information can be extinguished. Even though the concepts described above require more scientific substantiation, it can be concluded that the basic concepts of biophysical medicine are supported by the current state of science.

The Principles of Bioresonance Therapy

Bioresonance equipment is based on common electronic principles and is designed to process electromagnetic information [38]. Processed electromagnetic signals can be amplified or attenuated. Using electronic inversion filters, it is possible to shift the phase of the vibrations by 180° such that they are inverted. As a source of vibrations to be processed, electromagnetic information from the patient can be collected from the body, for example, using an electrode placed in the vicinity of a diseased organ. Furthermore, external electromagnetic information can be used for example from medicines, toxins, allergens, and gemstones. The primary principle of bioresonance therapy in eliminating pathological information within the body is the use of inverse information. When an electronically inverted vibration and the original vibration meet, both become extinct. Practical experience with this therapy shows that, in many cases, with the application of inverted vibrations several times for a few minutes, it is possible that relevant pathological information can no longer be traced to the patient. In many cases, this appears to be accompanied by partial or even complete disappearance of symptoms. Ultimately, it involves restoring the free flow of healing information or cell communication to support patients’ self-regulation and self-healing powers. In a review article, Foletti et al. [4] elaborated on these phenomena in bioelectromagnetic medicine.

The Practice of Bioresonance Therapy

The bioresonance equipment used in this study offers the option for each patient to receive individual treatment. This means addressing the needs of the patient using the patient’s own information and the possibility of testing and applying specific wave patterns from external sources. Various frequencies, from external sources or directly obtained via an electrode placed on the region of a diseased organ, are used as input information and subsequently as therapeutic information administered to the patient via output electrodes. For all the participants in this study, the gallbladder was the pathological organ that appeared to have a disturbed frequency pattern. The oscillations of the diseased gallbladder (‘cholecystitis’) together with the frequency pattern of the microbial invader (Salmonella) are considered pathological information. This information was administered in inverted mode to the patient over a few minutes using an output electrode. The specifications of this output information are determined using a test instrument called the biotensor, which is based on radiesthetic diagnostics.

The Biotensor

Biophysical research has shown that bioenergy has an unimaginably low signal intensity, which means that it is only possible to demonstrate these signals with the most advanced technology [31]. Likewise, biophysical research has shown that biological systems are sensitive to electromagnetic vibrations that are billions of times greater than those of the most advanced technical systems [39]. Therefore, unlike technical systems, biological systems are sensitive to bioenergy detection. Radiesthesia is based on this characteristic, and their testing methods are, therefore, common in biophysical medicine. Various energetic test methods based on these phenomena are described in the book ‘Geneeswijzen in Nederland’ [40]. This study used an instrument termed ‘biotensor’, which consists of a handle with a metal wire attached to it at the end of which was a metal cup- or ring-shaped object. Of the several diagnostic test methods that are possible using the biotensor, as described by Oberbach [41,42], the 'beziehungstest' (relationship test) was used in this study. Owing to their electronic circuitry, bioresonance devices can be used to amplify or invert the information transmitted from test ampules. During the measurement, the biotensor was placed vertically between the output signal of the bioresonance equipment and that of the patient. The biotensor indicates whether there is a positive or negative relationship between the two 'information systems'. For example, a clear relationship between a patient and an ampoule with cholecystitis information using an inverting module aimed at the patient is indicative of gallbladder inflammation.

## Materials and methods

Study design

The medical records of 41 patients who attended our clinic between March 2014 and January 2021 were reviewed (Table 1). Information was obtained from routinely collected health data for administrative and clinical purposes, without specific a priori research goals.

**Table 1:** Baseline characteristics of the patients. * IQR = Interquartile range ** CSC = Chronic Salmonella cholecystitis *** The time between the start of the biophysical treatments until the moment of the EOT measurement. EOT is the time at which the patient tests negative for all information in Table 2.

	Number	Percentage	Median	IQR*
Gender				
Male	13			
Female	28			
Age				
≤ 15	11	27		
16-25	17	41		
26-35	2	5		
36-45	4	10		
46-55	2	5		
≥ 56	5	12		
Duration of CSC**-complaints until intake in CAM practice (in months)			7	3-12
1-3	14	36		
4-12	15	38		
> 15	10	26		
Missing	2			
Treatment period (in months)***			1.5	1.2-2
≤ 1	11	27		
1,1-2	21	51		
2,1-3	7	17		
> 3	2	5		
Number of bioresonance treatments			4	3-5
1-3	17	42		
4-6	21	51		
7-9	3	7		
Time to follow up (in weeks)			20	6-60
1-10	12	33		
11-20	8	22		
21-30	5	14		
> 30	11	31		

Inclusion criteria

Criterion 1: All patients in the study group had persistent complaints of the upper gastrointestinal tract. 

Criterion 2: All patients had positive biophysical test results for all five information ampoules (Table 2) [43]. Within biophysical medicine, a test is considered positive when a resonance phenomenon can be demonstrated between the patient and the inverted pathological information. This could be demonstrated in all patients with the information from a diseased gallbladder (cholecystitis) and Salmonella. This meant that it could be assumed that there was gallbladder pathology and a salmonella infection. The test findings were supported by a positive test result for all other information ampoules as mentioned in Table 2 and tested with a non-inverted mode.

**Table 2:** Table 2. Overview of the information stored in a carrier substance (ampoule), developed and marketed by M. Keymer****. * This concerns circuits of bioresonance equipment in which the oscillation pattern is transmitted unchanged. ** This concerns circuits of bioresonance equipment in which the oscillation pattern is transmitted in an inverted mode. *** Salmonella-TP contains information on Salmonella typhi and Salmonella paratyphi. **** Therapeutisches Haus Martin Keymer, Bahnhofstraße 28, D-22941 Bargteheide.

Ampoule	Series	BICOM-program
Gallbladder	Attenuation ampoules	192/198*
Cholecystitis	Stomach, intestinal and liver pathology	191/197**
Gallbladder 2	Organ ampoules	192/198
Salmonella TP***	Bacteria	191/197
Gallbladder Meridian	'Five Elements'	192/198

Assessment and analysis

To assess the treatment results as objectively as possible, two external assessors in addition to the assessment of the author were included. They were given access to the anonymised medical records and asked to interpret the degree of self-reported improvement or worsening of complaints and assign a participant to one of the five groups at two points in time according to the assessment scheme in Table 3.

**Table 3:** Assessment of change of complaints, reported by patients.

Group	Reported change	Expressed as percentage	Hindering residual complaints
Group I	Deterioration	-	++++
Group II	No change	-	+++
Group III	Some improvement, but not satisfied	< 60	++
Group IV	Reasonable or sufficient improvement	60-80	+
Group V	Good or substantial improvement	80-100	+/-

The assessors were blinded to the assessment results of the other physician assessors. The first assessment was performed shortly after the information in question, as initially tested to meet the inclusion criteria could no longer be measured after several bioresonance treatments. The scores of this measurement moment were called ‘end-of-treatment’ scores (EOT). A few weeks later, depending on the possible comorbidities requiring treatment, a second assessment was performed. The scores of this measurement moment were called ‘Follow Up’ (FU) scores. The results were statistically analysed to determine the degree of agreement. To assess the reliability of outcome measures between the three assessors, intraclass correlation coefficients (ICCs) were computed using a two-way mixed model with an absolute agreement [44]. To test the significance of the treatment results, a one-sample Wilcoxon signed-rank test was performed in comparison with a hypothesised median of two for both the end-of-treatment (EOT) and follow-up (FU) scores [45]. The diagnostic outcomes for primary care (PC), hospital care (HC), and university hospital care (UHC) were recorded based on anamnestic (patient) data.

## Results

**Figure 1:**
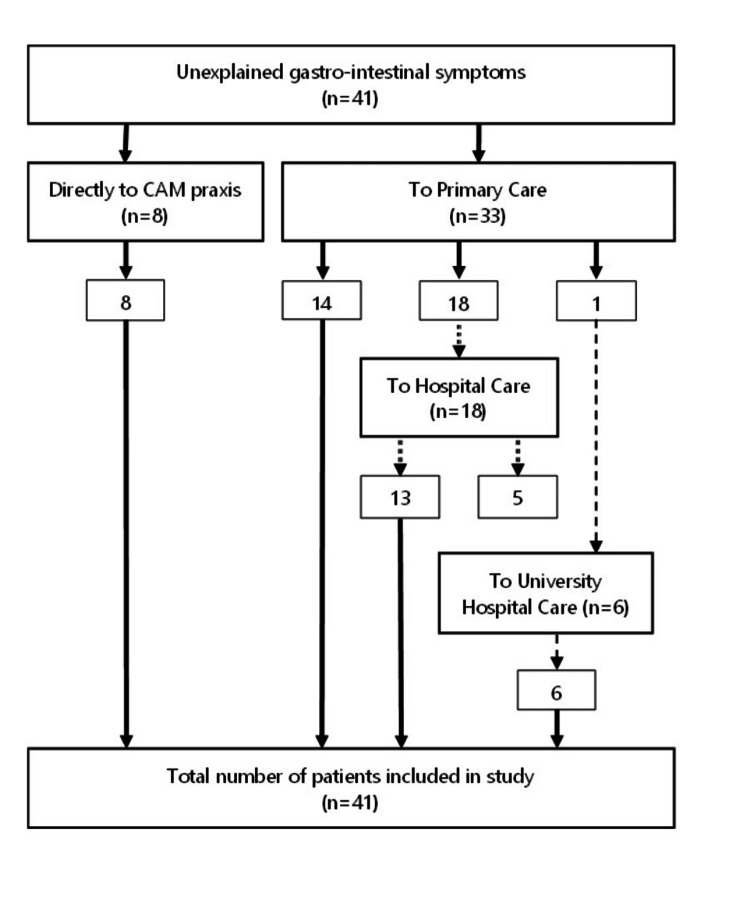
Flow diagram of the period before the patients registered in CAM practice

The flow diagram (Figure 1) shows that 80% of the patients went to the GP because of their complaints and that 57% of this group was referred to hospital care.

Tables 4-6 contain the explanations and/or diagnoses that the practitioners in PC, HC, and UHC gave to the patients before they reported to the CAM practice. These are copied verbatim into their files. It shows that the pattern of complaints did not lead to suspected gall bladder pathology (with one exception) but to a wide range of other possible causes of disease.

**Table 4:** Diagnostic outcomes of primary care (PC).

PC (n=33)	
Data missing or no results available	16
Low in vitamin D	2
Somatic inexplicable physical complaint	1
Gastritis	1
Functional dyspepsia	1
Problems with digestion	1
Constipation	1
Bad cooperation intestines	1
Food intolerance	1
Suspicion of gallstones	1
Irritable bowel syndrome	1
Gastro enteritis, post infectious	1
Fatty liver disease	1
Streptococcal infection	1
Viral infection	1
Morbus Pfeiffer	1
Adverse effect antiepileptic medication	1

**Table 5:** Diagnostic outcomes of hospital care (HC).

HC (n= 18)	
Somatic unexplained physical complaint	5
Irritable bowel syndrome	3
Obstipation	3
Parasite infection	2
Psychosomatic	1
Functional dyspepsia	1
No results available	3

**Table 6:** Diagnostic outcomes of university hospital care (UHC).

UHC (n=6)	
Somatically unexplained physical complaint	1
Endometriosis	1
Functional dyspepsia	1
Delayed gastric emptying	1
Rumination	1
No diagnosis communicated with patient	1

Analysis of the symptoms (Table 7) shows that nausea plays a role in most patients (93%). In this study, nausea should be considered a symptom with a high sensitivity for FGD/CSC. With the exception of vomiting, most of the other symptoms are unspecific and consistent with a wide range of other gastrointestinal diagnoses.

**Table 7:** Analysis of symptoms (n=41). * 1 participant with missing information on the specific symptom.

Symptom	Percentage
Nausea*	93
Fatigue	78
Abdominal pain	71
Weight loss	49
General malaise	32
Change of defecation pattern	28
Vomiting	20

The EOT and FU data from one patient were lost and subsequently excluded from the analysis. For two patients, the EOT scores could not be determined since relevant data were not available. These patients were excluded from the analysis. Two other patients were excluded based on their FU scores for the same reason. Therefore, in both outcome measures, 38 out of 41 patients participated in the analysis of the results (Table 8).

**Table 8:** Results of end-of-treatment (EOT) and follow-up (FU).

Results by group	End-of-treatment n (%)	Follow-up n (%)
1 worsening	0	0
2 no change	5 (13)	3 (8)
3 insufficient improvement	8 (21)	2 (5)
4 moderate improvement	13 (34)	7 (18)
5 good improvement	12 (32)	26 (68)
Total	38	38

The treatment results show that a total of 86% of the patients had a moderate or good improvement at follow-up. None of the patients experienced worsening of their symptoms during or after the biophysical treatments. This suggests that treatment is not associated with significant adverse effects.

In this study, 86% of patients showed improvement. Moderate improvement was achieved in 18% and good improvement in 68% of patients (median time to follow-up 20 weeks). Three patients reported a relapse of complaints. The patients underwent a second range of bioresonance treatments, and all three improved. The second treatment period was excluded from this study.

The one-sample Wilcoxon signed-rank test, a statistical validity score, showed a p-value of <0.001, for both end-of-treatment and follow-up scores. This suggests a high treatment efficacy of biophysical interventions. In addition, these interventions are non-invasive and inexpensive and have no side effects.

Statistical analysis

For the 'end-of-treatment' results (EOT), an ICC between the assessors of 0.675 (single measure) with a significance level of p<0.001 was found. For the follow-up results (FU), an ICC of 0.838 (single measure) was found, with a significance level of p<0.001. To evaluate the degree of improvement, a one-sample Wilcoxon signed-rank test was performed in comparison with a hypothesised median of 2 and showed a p-value of <0.001, for both the EOT and FU scores.

## Discussion

Diagnosis

As previously mentioned, several (partly) overlapping diseases, such as biliary dyskinesia, gallbladder spasms, chronic acalculous cholecystitis, chronic acalculous gallbladder dysfunction, and cystic duct syndrome, fall under the diagnosis of FGD. They are considered synonymous and have dysmotility of the gallbladder as a central factor in their pathogenesis. It has become clear in the foregoing that FDG in conventional medicine cannot be defined according to strict diagnostic criteria, although this has been attempted over the years. An important supportive Rome-4 criterion is a low ejection fraction of the gallbladder measured using cholescintigraphy (<40%).

Previous studies show that this cannot be maintained in practice [14]. Demonstrable gallbladder motility dysfunction is an important but not mandatory criterion for FGD as the diagnosis is made in cases where scintigraphy shows no abnormalities [46,47]. In other cases, the FDG diagnosis is solely based on associated complaints according to the Rome 4 criteria [48]. Patients with atypical symptoms of biliary colic who do not meet these criteria are excluded from the diagnosis. Hansel et al. [13] concluded that the diagnosis and management of FGD in conventional medicine remains a puzzling exercise. There is a need for consensus on the symptoms defining biliary dyskinesia and validation of the testing required for its diagnosis of biliary dyskinesia [49]. This study shows that biophysical medicine offers new, consistent, and effective perspectives regarding the diagnosis of FGD. The study uses a clear and strict inclusion criterion: only patients with persistent and unexplained upper abdominal complaints (Table 5) with five positive biophysical findings (Table 2) were included in the study. The study concludes that the diagnosis in a significant share of these patients is based on a bioenergetically definable chronic gallbladder inflammation, in conjunction with a Salmonella burden, termed 'chronic Salmonella cholecystitis'. Furthermore, it can be assumed that the biophysical diagnosis of CSC falls under the clinical syndrome of FGD, but based on completely different criteria.

Diagnostic gap

This study also revealed that the combination of complaints in question was not exceptional among the Dutch, and possibly even the European, population. This contrasts with the frequency at which the diagnosis of FGD is established in the conventional trajectory. None of the patients in this study were diagnosed with FGD. In the previous medical trajectory, gallbladder pathology was suspected in only one patient. This makes it highly probable that the majority of patients who qualify for the diagnosis of FDG based on their symptoms are not diagnosed as such. Complaints are usually ‘medically unexplained physical symptoms' or other diagnoses (Table 4). This was true for all the 41 patients who participated in this study. One explanation for this diagnostic gap may be that little or no attention has been paid to functional gallbladder complaints, at least in the Dutch medical curriculum. A second likely reason is that the pathology of the gallbladder is generally associated with older age, whereas 68% of the patients in the study group were younger than 25 years. A Dutch textbook of paediatrics [50] does not mention functional gallbladder disorders; however, 27% of the patients in the study group were 15 years or younger.

Energetic-informative disorder of the gallbladder

‘Cholecystitis’ implies inflammation. More advanced inflammation is macroscopically characterised by morphological changes detectable using diagnostic imaging techniques, which, in conventional medicine, are applied almost invariably in all patients with related complaints. No morphological or microbiological abnormalities were observed in the study group during the preliminary clinical research trajectory. The question that arose was whether the absence of morphological changes precluded ‘cholecystitis'. The unambiguous answer to this question from a complementary medicine perspective is that it does not. The same phenomenon was observed in a more stringent study by Roumen et al. [51]. In this study, the expected correlation between chronic appendicitis pain scores and relevant histological findings could not be confirmed. Histopathological examination revealed that nearly 50% of the excised appendices showed no signs of inflammation. This paradox has also been observed by Jones et al. [52]. In this study, the histopathological results were inconclusive. After cholecystectomy, a clear symptom reduction was observed in 36 patients with biliary dyskinesia. The initial histopathological examination of the preparations showed signs of chronic inflammation in 83% of the cases, whereas, in the retrospective judgement preparations, significant chronic inflammation dropped to 38%. Both examples show that the disease can exist without histopathological signs of inflammation in the affected organs. This can be regarded as the core concept of complementary medicine. An essential distinction between mainstream and complementary medicine is that the former focuses on anatomical, morphological, and functional abnormalities. Somewhat exaggeratedly, it can be stated that conventional medicine can only make a somatic diagnosis if the disease has developed to such an extent that structural abnormalities can be detected with diagnostic techniques such as X-ray, CT-scanning, MRI, biopsies, and others. In contrast, the diagnostic methods that complementary medicine has developed over time are invariably characterised by a focus on the immaterial or energetic stages of the disease. In this study, only one patient reported suspected gallbladder pathology. It can be assumed that, in the other patients who underwent a diagnostic process, neither morphological changes in the gallbladder nor microbial pathogens could be detected. In contrast, in the study group, diagnoses were made based on energetically informative principles. Without exception, CSC can be diagnosed in this manner. Therefore, in biophysical medicine, CSC is regarded as an energetically informative disorder of the gallbladder caused by pathological Salmonella oscillations.

This involves electromagnetic vibrations, which are thought to persist for months or years after a Salmonella infection. This condition maintains a subclinical form of inflammation but rarely develops into fulminant cholecystitis. Contamination with Salmonella is considered the primary cause, with the idea being that the causative bacteria may have been eliminated some time ago, while the pathological oscillations of the causative agent are still persistent, often leading to dysregulation of gallbladder motility. The inclusion criteria used in this study and the treatment results suggest that the condition described as a Salmonella-induced FGD is a true clinical entity. In the author’s opinion, this entity can be responsible for a wide range of nonspecific and often misunderstood upper abdominal complaints.

Results

Currently, patients with FGD-like complaints often remain in conventional medicine. The analysis of our patient group showed that the complaints were not properly understood or interpreted and that no effective treatment could be provided. There is consensus that laparoscopic cholecystectomy is the treatment of choice for these patients. However, it remains an intervention with risks and possible residual complaints. Mahid et al. found the 'medical watchful waiting' approach questionable and often results in insufficient symptom reduction [53]. It can be concluded that both approaches produce questionable treatment results [50].

The results of the current study, in which 86% of participants reported fair to good improvement, are consistent with the review studies by Santucci et al. [49] and Mahid et al. [53]. Santucci et al. [49] showed that laparoscopic cholecystectomy produces a success rate ranging from 34% to 100%. Mahid et al. [53] concluded in a meta-analysis that 96% of patients (n=462) reported some degree of symptom relief after surgery and only 45% reported comparable symptom relief with medical treatment (n=153). The results of the current study do not provide evidence for the effectiveness of the biophysical approach to FGD. They do offer arguments for no longer approaching the condition FGD exclusively surgically and invite us to further study the biophysical approach as a valuable treatment option.

This recommendation is consistent with the SIO Clinical Practice Guideline Model [54]. The risk-benefit analysis appears to be strongly in favor of both biophysical diagnosis and biophysical treatment. The study, thus, supports the statement of the Italian physician and researcher Foletti [4] when he wrote that the recognition and application of the principles of biological electromagnetism will lead to significant progress in medicine.

Limitations of the study

Patients came to the author's practice on their own initiative, often based on the recommendations of family members or friends. There is a source of bias in this, as well as the fact that patients must bear part of the costs themselves (selection bias). This observational retrospective study has some other limitations. Data were derived from patient files without the intention of using them for publication at a later stage. No validated questionnaires were administered. As previously indicated, the patients were not referred to our practice but often came on their own initiative; therefore, information regarding their medical history was limited and partly dependent on what the patient could remember (recall bias). Previous conventional medical diagnoses were recorded as reported by the patient. A number of the participating patients had comorbidities, such as post-viral syndromes (six), borreliosis (three), premenstrual syndrome (one), nutritional intolerance (five), and gluten enteropathies (six), which obscured, to some extent, the clinical picture and treatment results.

Strength of the study

The effects obtained are likely generalizable because this research did not take place in a research setting but in an 'ordinary' CAM practice with a biophysical treatment method that can be taught to any healthcare professional. Moreover, the strength of the study is the fact that strict objective inclusion criteria were used, data were rarely missed, and there were virtually no losses to follow-up (of the 41 patients included, the treatment results of 38 were analysed). Finally, the study was of high quality because the degree of change in complaints was measured at two time points by three assessors in a blinded setting.

## Conclusions

The diagnosis of the patients in this study was based on a bioenergetically definable chronic gallbladder inflammation arising in conjunction with Salmonella burden, termed CSC, which must be considered as a true clinical entity. CSC should be considered an energetic-informative disorder of the gallbladder caused by pathological Salmonella oscillations that may lead to persistent dysregulation of gallbladder motility. It can be assumed that the biophysical diagnosis of CSC falls under the clinical syndrome of FGD based on completely different diagnostic criteria.

It is highly probable that the majority of patients who qualify for a diagnosis of FGD based on their symptoms are not diagnosed as such. In this study, 86% of the patients had fair to good treatment outcomes. Therefore, a biophysical approach to CSC/FGD appears to be a viable treatment option when costs, benefits, risks, and side effects are considered.
